# Supplementary material for: Universal attenuators and their interactions with feedback loops in gene regulatory networks
Source: Nucleic Acids Res. 2017 Jun 1;45(12):7078–93. doi: 10.1093/nar/gkx485 (PMC5499555; doi:10.1093/nar/gkx485)
Supplement: Supplementary Data [file gkx485_Supp.docx]

Supplementary material

# List of Abbreviations:

Transcription factor: TF

Gene regulatory network: GRN

Linear regulatory chain LRC

Relative effectiveness: RE

Lowest level of expression: LLE

Highest level of expression: HLE

Mean position across all LRCs: MPAL

## Selection of model

Transcriptional regulation plays a key role in controlling the behaviour of cells and many modelling approaches have been used to explore the different facets of its biology. Among the models built for this purpose, it is possible to find methods ranging from ordinary or partial differential equations to deterministic or stochastic Boolean networks (1,2,3). When ordinary differential equations are used, the interactions between a transcription factor and a gene assumes a mathematical form that is closely related to the one that we used.

In particular, it is generally possible to observe 1) a switch-like behaviour, which is due to an activation threshold, 2) saturation at a high concentration of the TF (related to HLE), which is due to competitive binding, 3) a baseline level of transcription at a low level concentration of the TF, which is due to non-specific TF bindin, and 4) a Hill coefficient – which models the cooperativity of the TFs. The rationale for these features is grounded in the biology of transcriptional regulation and biological evidence supporting the functional structures being used (1).

The ordinary differential equation model that we chose allows an extensive parameter exploration that would be unachievable with more complex modelling approaches. Moreover, TF expression is crucial to maintain the appropriate transcriptional programs of cells and therefore it is plausible to assume that their expression is significantly larger than zero, hence satisfying the general conditions of a deterministic differential equation system. Nonetheless, it is possible that stochastic effects may play a significant role in single- and multi-step transcriptional regulation and future work will be devoted to explore this aspect.

## Linear chains in other systems

Our work was focused on the effect of linear chains of regulation in the context of transcriptional regulation. However, similar chains have been encountered and studied in other systems. For example, it has been found that long signalling cascades can reduce noise of signals (4). It has also been suggested that ecological systems possess a limited number of trophic levels (and hence short chains in the food web) due to the limited amount of energy that can flow from one level to the other. Finally, after the seminal experiment of Stanley Milgram, it has been consistently found that even in social networks short chains are very common, leading to the famous concept of “six degrees of separation”. In all of these examples it is possible to identify a directional mode of interaction that is akin, at least conceptually, to the linear chains of regulation that we have studied. This suggests that the mechanisms that we explore in our current work may be playing a role outside of transcriptional regulation.

## Supplementary figures

Figure S. 1. Properties of simulated LRCs. (A) Effect of parameters on regulatory mode. Example regulatory functions are plotted for different parameters in Equation 1 of the main text. When β>α the function describes a transcriptional activator (curves 1 and 2). When β=α,the function describes a transcriptional interaction that neither activates nor inhibits (curve 3). When β<α the function describes a transcriptional inhibitor (curves 4 and 5). (B) RE for LRCs of activators. The same conventions of Figure 2B apply. Note the clear exponential decrease (C) RE for LRCs of mixed repressors and activators. The same conventions of Figure 2B apply. Note the clear exponential decrease. (D-G). Example response functions are reported when only one parameter of Equation 1 is independently varied. Larger values of 𝛼 result in a higher concentration of the target gene in the absence of transcriptional regulation (D). Larger values of *β* result in a higher concentration of the target gene at high concentration of the transcriptional regulator (E). Larger values of *h,* the Hill coefficient, describe a higher “binding cooperativity” of the transcriptional regulator and result in a sharper transcriptional response (F). Larger values of *s* result in a detectable transcriptional response at lower concentrations of transcriptional regulator.

Figure S.2. Combinatorial exploration of the effect of variation in the parameters characterising Equation 1 on relative effectiveness. (A-C) The adjusted *R*^2^, *p*-value for the slope, and *d*, are plotted against the maximum of the sampling range for α and β, when the minimum is fixed to 0, with boundaries excluded. Each point indicates the average of 1000 simulations when *h* is fixed to 1 and *s* is fixed to 2. The adjusted *R*^2^ is consistently high (A), indicating that a decrease describes very well the behaviour of RE. Moreover, the *p*-value associated with the slope is consistently low (B), indicating an exponential decrease significantly different from zero. Nonetheless, a significant variation in the value of *d*, indicating the strength of the exponential decrease can be observed (C). Taken together, these results indicate that a statistically significant exponential decrease can be observed, but that, unsurprisingly, the magnitude of the decrease is affected by the actual variation in the parameter ranges of α and β. (D-F) The same information of Panels A-B is reported against the maximum of the sampling range for *h* and *s*, when the minimum is fixed to 0, with boundaries excluded. Each point indicates the average of 1000 simulations when α is fixed to 10 and β is fixed to 0.01.Note the comparable conclusions for the adjusted *R*^2^, the *p*-value for the slope and the value of *d*.

**Table S.1. Functions of the transcriptional regulators encountered in the longest LRCs of *E.coli* (data adapted from EcoCyc 2014) (5, 6)**

Figure S. 3. Additional information for *E. coli* and *M. tuberculosis*. (A) Potentially chaotic feedback loops in the GRN of *E. coli.* As described before, only two feedback loops composed by three or more genes can be found in the GRN of *E. coli* (7). These feedback loops are potentially chaotic. (B) Potentially chaotic feedback loops in the GRN of *M. tuberculosis.* Only two three-node feedback loops can be found in the GRN of *M. tuberculosis* (7). Note how they share the same configuration as the chaotic motifs of *E. coli.*

Figure S. 4. Distribution of the length of LRCs in different organisms and cell types*.* The number of LRCs of different lengths is reported. Length distribution of the real network is in blue and those for average rewired (randomised) and random networks are in brown and red respectively. Organisms/cell types from A to E are *E. coli* K12, *M. tuberculosis, S. cerevisiae*, human GM12878 and K562 cells.

1. Hooshangi S, Thiberge S, Weiss R. Ultrasensitivity and noise propagation in a synthetic transcriptional cascade. Proc Natl Acad Sci U S A. 2005;102(10):3581-6.

2. Xiao Y. A tutorial on analysis and simulation of boolean gene regulatory network models. Curr Genomics. 2009;10(7):511-25.

3. Sanchez A, Choubey S, Kondev J. Stochastic models of transcription: from single molecules to single cells. Methods. 2013;62(1):13-25.

4. Thattai M, van Oudenaarden A. Attenuation of noise in ultrasensitive signaling cascades. Biophys J. 2002;82(6):2943-50.

5. Keseler IM, Collado-Vides J, Santos-Zavaleta A, Peralta-Gil M, Gama-Castro S, Muniz-Rascado L, et al. EcoCyc: a comprehensive database of Escherichia coli biology. Nucleic acids research. 2011;39:D583-D90.

6. Keseler IM, Mackie A, Peralta-Gil M, Santos-Zavaleta A, Gama-Castro S, Bonavides-Martinez C, et al. EcoCyc: fusing model organism databases with systems biology. Nucleic acids research. 2013;41(D1):D605-D12.

7. Albergante L, Blow JJ, Newman TJ. Buffered Qualitative Stability explains the robustness and evolvability of transcriptional networks. eLife. 2014;3.
